# Lactic Acid Bacteria: embarking on 30 more years of research

**DOI:** 10.1186/1475-2859-13-S1-I1

**Published:** 2014-08-29

**Authors:** Jan Kok, Eric Johansen, Michiel Kleerebezem, Bas Teusink

**Affiliations:** 1 Department of Molecular Genetics, Groningen Biomolecular Sciences and Biotechnology Institute, University of Groningen, Top Institute Food and Nutrition, Wageningen the Netherlands; 2 Innovation, Chr. Hansen A/S, Hørsholm, Denmark; 3 Host-Microbe Interactomics Group and Top Institute Food and Nutrition, Wageningen, NIZO Food Research, Ede, the Netherlands; 4 Systems Bioinformatics, Amsterdam Institute for Molecules, Medicines and Systems, VU University Amsterdam, the Netherlands

## 

The 11^th ^International Symposium on Lactic Acid Bacteria

Lactic Acid Bacteria play important roles in the production of food and feed and are increasingly used as health-promoting probiotics. The incessant scientific interest in these microorganisms by academic research groups as well as by industry has resulted in many significant scientific breakthroughs and has led to new applications. A series of tri-annual symposia on Lactic Acid Bacteria was started in 1983 in order to communicate and stimulate research on Lactic Acid Bacteria and their application. These symposia have allowed researchers from academia and industry to meet, present their work and be informed in an international, open and pleasant atmosphere. Thus, the LAB symposia have, over the years, always presented the state-of-the-art in the field. We have over the past decades witnessed a tremendous increase in our understanding of the basic physiology and genetic make-up of these microorganisms and, from the turn of the millennium onwards, have been able to apply all 'omics' techniques in order to get in-depth understanding of the molecular biology and application potential of lactic acid bacteria.

Now, in 2014, we are looking forward to embarking on the next phase and to set the stage for another 30 years of research on this industrially highly relevant group of bacteria. Focus will be on their eminent importance in health and nutrition of humans all over the world. The breadth and richness of LAB research is exemplified by the six selected scientific areas that will be covered at the LAB11 Symposium: Diversity and Evolution; Genetics and Physiology; Ecosystems and Sustainability; Fermentation and Industrial Application; Host-Microbe Interactions; and Emerging Technologies. Leading, as well as upcoming LAB scientists have been selected to cover the latest developments in these research areas, while renowned scientists from outside the LAB-community have been invited to cover important emerging fields.

For all 11 LAB lectures presented at the LAB11 symposium the authors have prepared review-type manuscripts. These have gone through a standard peer review process, for which we were invited to act as Guest Editors for Microbial Cell Factories. These papers are presented here, online and in a printed version as the "Symposium Proceedings" for all who attend the meeting in Egmond aan Zee. We are obliged to the MCF Editor-in-Chief, dr. Antonio Villaverde, to Isobel Peters at BioMed Central and to all our authors and reviewers for the smooth editorial process. We wish you good reading.

## Competing interests

The authors declare that they have no competing interests.

## Declarations

This article has been published as part of *Microbial Cell Factories *Volume 13 Supplement 1, 2014: Proceedings of the 11th International Symposium on Lactic Acid Bacteria. The full contents of the supplement are available online at http://www.microbialcellfactories.com/supplements/13/S1. Publication charges for this supplement were funded by the Stichting Symposium on Lactic Acid Bacteria. The articles have undergone the journal's standard peer review process for supplements. EJ is employed by Chr Hansen A/S, is a shareholder in Chr Hansen A/S; and has applied for patents relating to the content of an article which he did not edit but which forms part of this supplement. MK is an employee of Nizo. The other Supplement Editors declare that they have no competing interests.

